# Rubber-enhanced polyamide nanofibers for a significant improvement of CFRP interlaminar fracture toughness

**DOI:** 10.1038/s41598-022-25287-y

**Published:** 2022-12-11

**Authors:** Emanuele Maccaferri, Matteo Dalle Donne, Laura Mazzocchetti, Tiziana Benelli, Tommaso Maria Brugo, Andrea Zucchelli, Loris Giorgini

**Affiliations:** 1 Department of Industrial Chemistry “Toso Montanari”, University of Bologna, Viale Risorgimento 4, 40136 Bologna, Italy; 2 Interdepartmental Center for Industrial Research on Advanced Applications in Mechanical Engineering and Materials Technology, CIRI-MAM, University of Bologna, Viale Risorgimento 2, 40136 Bologna, Italy; 3 Department of Industrial Engineering, University of Bologna, Viale Risorgimento 2, 40136 Bologna, Italy

**Keywords:** Composites, Mechanical properties, Polymers, Mechanical engineering

## Abstract

Nanofibrous mats provide substantial delamination hindering in composite laminates, especially if the polymer (as rubbers) can directly toughen the composite resin. Here, the well-known Nylon 66 nanofibers were impregnated with Nitrile Butadiene Rubber (NBR) for producing rubber/thermoplastic membranes for hampering the delamination of epoxy Carbon Fiber Reinforced Polymers (CFRPs). The starting polyamide mats were electrospun using two different solvent systems, and their effect on the mat's thermal and mechanical properties was investigated, as well as the laminate Mode I delamination resistance via Double Cantilever Beam (DCB) tests. Plain Nylon 66 mats electrospun from formic acid/chloroform perform better than the ones obtained from a solvent system containing trifluoroacetic acid, showing up to + 64% vs + 53% in interlaminar fracture toughness (G_I_), respectively. The effect of NBR coating benefits both nanofiber types, significantly raising the G_I_. The best results are obtained when interleaving medium-thickness and lightweight mats (20 µm, 9–10 g/m^2^) with 70–80 wt% of loaded rubber, achieving up to + 180% in G_I_. The work demonstrates the ability of NBR at improving the delamination hindering of common polyamide nonwovens, paving the way to the use of NBR-coated Nylon 66 nanofibers as effective interleaves for G_I_ enhancement and overall composite safety improvement.

## Introduction

Composite materials represent the best choice to obtain structures with outstanding mechanical properties. In particular, Carbon Fiber Reinforced Polymer (CFRP) laminates are progressively replacing, where possible, metallic materials to benefit from improved lightness. Despite many advantages, such as high specific modulus and strength, corrosion resistance, helping fuel saving, and easiness of production, composite laminates suffer from some relevant weaknesses. Delamination is undoubtedly the most severe drawback affecting such materials, leading to complete component failure with potentially catastrophic consequences. The reduction of delamination risk is crucial for allowing further applications of composite laminates in fields currently precluded due to reliability and safety concerns. Moreover, the improved delamination resistance raises the overall composite sustainability by potentially increasing the component lifetime. Any laminate is susceptible to delamination because of its intrinsic anisotropic 2D-like stack structure, which is responsible for the reduced mechanical performance between laminae. Even though several strategies may be implemented for monitoring the health of a composite component, like the exploitation of Bragg fibers or piezoelectric materials (even nanostructured)^1–4^, these systems are expensive and, consequently, scarcely used in common applications.

Many simple and economical ways to avoid delamination involve matrix and/or interlaminar region modification to improve the fracture toughness. Since matrix properties govern the interlaminar behaviour, its modification may strongly affect the final composite performance; this often happens with bulk matrix toughening achieved by adding tougheners, like rubbers or suitable thermoplastic polymers. Regarding the modification with rubber, it can be an uncrosslinked “liquid” rubber or crosslinked rubbery particles^5–9^. While this type of modification is simple to attain, it involves a specific resin formulation. Moreover, the change interests the resin bulk and, in turn, the whole component, usually leading to lowered mechanical, thermal, and thermomechanical properties, besides a significant weight increase.

Localized modifications, instead, are smarter, allowing a targeted intervention only in the most critical regions, like interlaminar ones, where stress concentrations occur^10^. The potential benefits are many: retention or limited—and confined—lowering of the overall component’s thermal and mechanical properties, low increment of weight and dimension. Moreover, this type of modification can be virtually applied to any available commercial prepreg since the whole resin is not affected. The integration of bulk viscoelastic layers (films) between laminae^11–13^, still representing a localized, economical and straightforward solution, negatively affects laminate stiffness, strength, weight, and size^14^. Less impacting solutions have been practiced since the spreading of nano-reinforcements occurred. Indeed, they can be used to achieve the desired effects by adding low quantities^15–17^, thus benefitting from negligible composite size and weight changes. Adding nanoparticles^18,19^ and carbon nanotubes (CNTs)^20–23^ proved to boost the composite performance. However, in some cases, they are expensive and hard to handle.

Since the mid-90 s, electrospinning has been proposed as a versatile process to produce polymeric nanofiber nonwovens. In 2001, electrospun materials were used, for the first time, to reinforce composite laminates against delamination by interleaving nanofibrous mats between prepreg plies, allowing local modification in the interlaminar matrix-rich region^24^. Their integration, carried out during the lamination step, is simple compared to other nano-reinforcements, such as CNTs. Nanofibrous membranes can significantly enhance the interlaminar fracture toughness (G), that is, the energy per unit area required for the crack propagation^25,26^. Depending on the nanofibrous mat thermal properties, two main mechanisms might set in against crack propagation: (i) nanofiber bridging and (ii) matrix toughening. Thermoplastic polymers with a melting temperature, *T*_m_, (or a glass transition temperature, *T*_g_) above the composite curing cycle temperature maintain the nanofibrous structure in the final laminate. This type of nonwoven acts as bridging threads that help keep the diverging edges together (mechanism i)^26^. Instead, the matrix toughening (mechanism ii) occurs when fibers fluidize (i.e., with *T*_m_ for semicrystalline polymers or *T*_g_ for amorphous ones below the curing temperature) and blend with the continuous resin phase. Both mechanisms raise the energy required for the crack propagation. When dealing with nanofiber bridging, the crack to propagate needs to overcome the 3D network constituted by the nanofibrous mat. By contrast, in the other case (mechanism ii), the crack faces a less fragile matrix thanks to the toughening induced by the mixing of the thermoplastic polymer with the resin. It is worth pointing out that the chosen polymers should be compatible with the matrix: a good polymer-resin interaction at the interface is necessary for non-melting nanofibers, while miscibility for low-*T*_m_ polymers (or low-*T*_g_ for amorphous ones) is usually required.

While the integration of thermoplastic nanofibers is renowned and extensively applied^26,27^, the use of rubbery nanofibers is still not. Until now, only a few works have proposed the production of rubbery fibers, and most of them are just proofs-of-concept^28–30^. The difficulty in obtaining such nanostructures lies in the rubber cold flow, which prevents fibrous shape retention. Recently, the authors reported the possibility of producing uncrosslinked rubbery nanofibers made of nitrile butadiene rubber (NBR) via blending with poly(ε-caprolactone) (PCL)^31^. Their integration into epoxy CFRP laminates demonstrated a remarkable effect against delamination thanks to local matrix toughening, as evidenced by SEM micrographs of delamination surfaces^32^. Clearly, these nanofibers exclusively act via mechanism ii. By replacing PCL with the high-performance Nomex (PMIA, poly-*m*-phenylene isophthalamide), characterized by a *T*_g_ around 280 °C, it is possible to combine mechanisms i and ii^33^.

The simultaneous action of both mechanisms, that is, nanofiber bridging and matrix toughening, may help contrast delamination more efficiently. Some studies^34,35^ reporting the use of core–shell nanofibers made of Nylon 6 (inner phase) and PCL (outer phase) investigate the effect of the interdiffusion of the polyester into the resin, which can occur during the curing process. Indeed, while Nylon 6 melts above 200 °C, the PCL *T*_m_ is near 60 °C; thus, depending on the curing cycle temperature, it is possible to modulate the extension of PCL interdiffusion into the matrix. Results reveal a potential positive role of the fluidizable PCL component on the overall reinforcing effect.

In this frame, using rubber as an “interdiffusing material” instead of non-elastomeric thermoplastic ones may further improve the interlaminar fracture toughness. Moreover, even if valid, the use of core–shell nanofibers poses some limits, like difficulties in controlling and tailoring the ratio of inner and outer polymers, as well as a more complicated processing with respect to single-needle electrospinning.

In this work, the well-known Nylon 66 nanofibers were post-processed after the mat production to be impregnated with NBR for producing rubber/thermoplastic membranes for hindering delamination of epoxy CFRP composites. Different mat thicknesses (grammages) of the thermoplastic nanofibers, loaded with different amounts of uncrosslinked NBR, were investigated. The delamination resistance of the nanomodified laminates was assessed in Mode I via Double Cantilever Beam (DCB) tests, and compared with the unmodified CFRP. Moreover, the effect of two different Nylon 66 solvent systems on the mat thermal and mechanical properties, as well as on the final CFRP performance, were also investigated.

**Figure 1:**
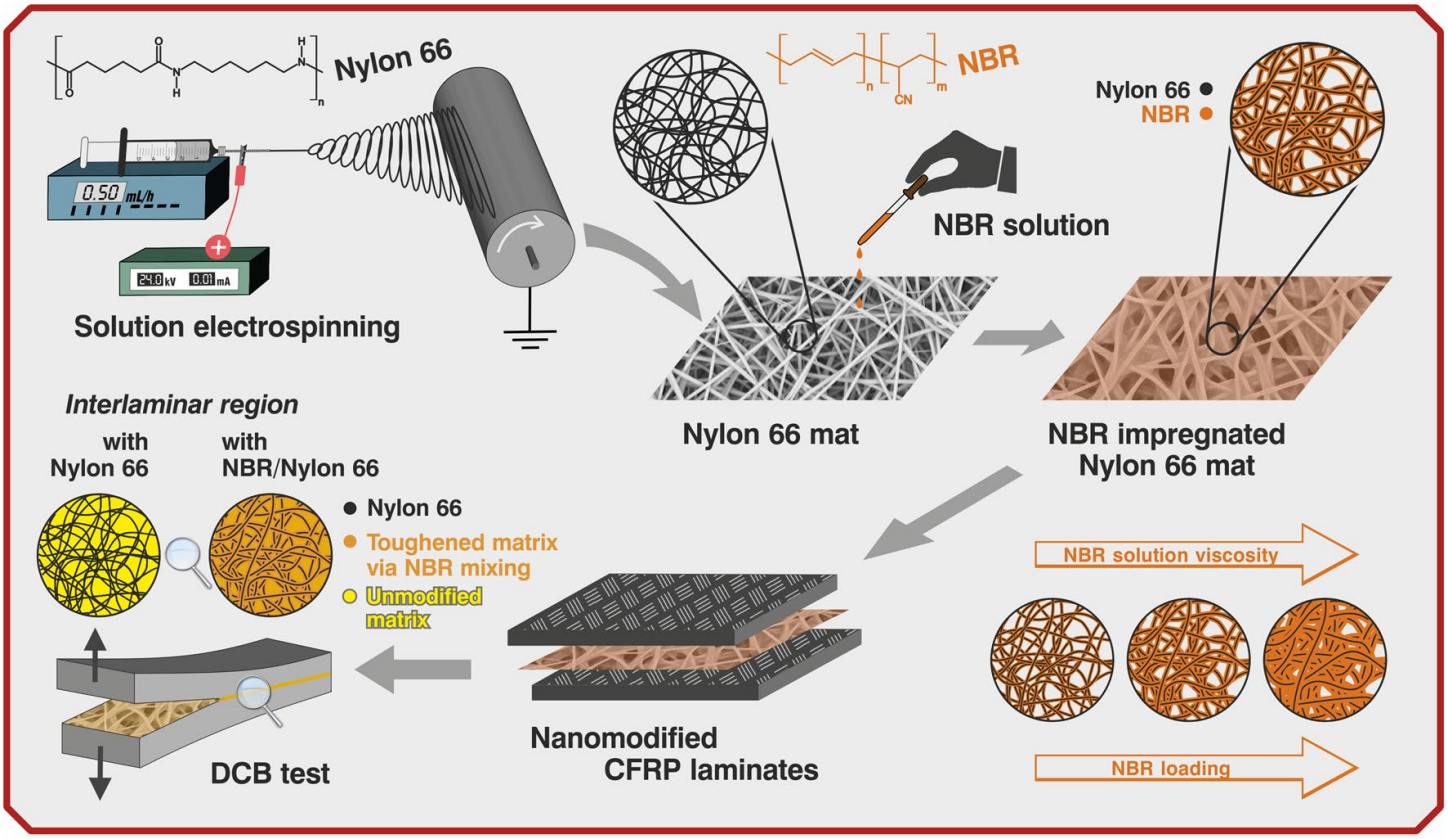
Overview of the work: electrospinning of Nylon 66 nanofibrous mat, its impregnation with nitrile butadiene rubber (NBR) solutions at different viscosities to obtain NBR/Nylon mats, and evaluation of the interlaminar fracture toughness of the nanomodified CFRPs via DCB test.

In Fig. 1 is depicted a sketch of the paper rationale.

## Results and discussion

The efficacy of nanofibrous mats, irrespective of the acting mechanism (nanofiber bridging and/or matrix toughening), at hindering delamination is well documented in the literature^26,36–39^. Previous works from the authors^32,40,41^ regarding the interleaving of nitrile butadiene rubber/poly(ε-caprolactone) (NBR/PCL) rubbery nanofibrous mats demonstrate a remarkable increase in the CFRP interlaminar fracture toughness and damping. This polymer pair acts exclusively via matrix toughening mechanism, as assumed from the polymers thermal properties, and confirmed by SEM delamination surfaces showing an extensive resin deformation and a ductile fracture. Indeed, NBR (*T*_g_ < *T*_amb_) and PCL (*T*_m_ ≈ 60 °C) may diffuse into the epoxy resin during the curing cycle, leading to a toughened matrix. It was also demonstrated that NBR adds a significant toughening ability to Nomex nanofibers that, on themselves, lead to poor composite interlaminar properties^33^, probably due to a negative interference with the crosslinking process^42^, besides a poor adhesion with the epoxy resin. In the cited cases, NBR/PCL blend and NBR/Nomex self-assembled mixed nanofibers were produced via single-needle electrospinning.

**Figure 2:**
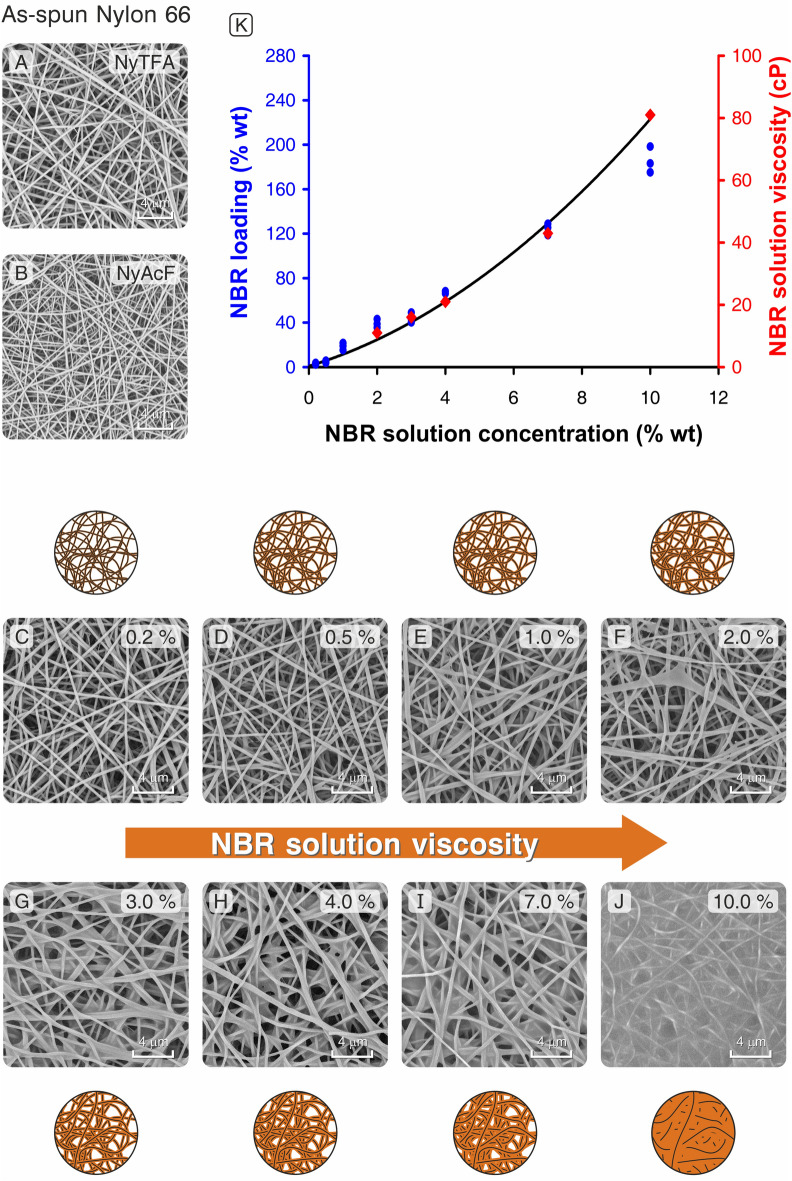
(**A**) NyTFA and (**B**) NyAcF SEM micrographs of as-spun Nylon 66 nanofibrous mats. (**C**–**J**) SEM micrographs of NBR impregnation tests on NyTFA mats: effect of different impregnating solution concentration. From (**C**) to (**J**): use of impregnating solutions at 0.2, 0.5, 1.0, 2.0, 3.0, 4.0, 7.0, and 10.0 wt%. (**K**) NBR loaded on Nylon 66 nanofibers and NBR solution viscosity as a function of NBR solution concentration. The impregnation tests were carried out on the NyTFA_40 mat, three repetitions. The data fitting made for viscosity measurements is a 2nd polynomial function; the interception with the y-axis was set at the value of the acetone viscosity (0.32 cP^43^). Viscosity measurements on 0.2, 0.5, and 1.0 wt% NBR solutions were not performed because of a too low viscosity medium.

Here, NBR solutions were applied as a post-manufacturing treatment onto Nylon 66 nanomats via hand impregnation to maximize the toughening effect by combining the nanofiber bridging and matrix toughening mechanisms. Such an approach bypasses the complicated core–shell methodology and the crucial issue of finding a common solvent for the two polymers in the case a single-needle procedure would be applied, as in the case of NBR/Nomex pair. The latter is indeed impossible with the NBR/Nylon 66 pair, since formic acid, an essential component for solubilizing the polyamide, is a complete non-solvent for the rubber precursor, causing instantaneous polymer precipitation. The plain polyamide nanofibrous mats were obtained using two different solvent systems, namely NyTFA and NyAcF. Moreover, the electrospinning process was adapted to attain different mat thicknesses comprised in the 3–25 g/m^2^ range, as reported in Tables 1 and S1. NyTFA membranes were produced from a solution with TFA/formic acid/CHCl_3_ 11:55:34 wt as solvent system, while NyAcF mats were electrospun from a formic acid/CHCl_3_ 1:1 wt solution. In both cases, SEM investigations (Fig. 2A,B) show a random deposition of nanofibers, as required to obtain an isotropic reinforcement in the laminate plane. NyTFA and NyAcF membranes are characterized by comparable fiber diameters: 259 ± 53 nm and 232 ± 44 nm, respectively.

**Table 1:** Mat characteristics before and after impregnation with NBR solutions at 3.0 wt% and 7.0 wt%.

Nanofibrous mat	Nylon mat grammage (g/m^2^)	Loaded rubber (wt%)		Ratio B/A
		(A) with 3.0 wt% NBR solution	(B) with 7.0 wt% NBR solution	
NyAcF_10	2.7 ± 0.3	80–100	250–300	≈ 3.1
NyAcF_20	5.2 ± 0.5	75–85	155–200	≈ 2.2
NyAcF_40	10.8 ± 0.7	45–55	115–130	≈ 2.5
NyTFA_10	3.1 ± 0.2	75–90	220–260	≈ 3
NyTFA_20	5.9 ± 0.5	60–75	175–215	≈ 2.9
NyTFA_40	11.4 ± 0.8	40–50	120–130	≈ 2.8

### Preliminary NBR impregnation tests of Nylon 66 mats

Several impregnation tests were carried out to investigate the effect of the NBR impregnation on the mat morphology and overall grammage. Since it is expected that the amount of loaded rubber should be related to the impregnating solution viscosity, NBR solutions with different concentrations—from 0.2 to 10.0 wt%—were tested. SEM micrographs in Fig. 2C–J show the morphologies obtained for NyTFA mats (NyAcF mats display similar morphologies).

By carefully choosing the concentration of the impregnating solution, it is possible to modulate the NBR loading onto the Nylon nanofibers (Fig. 2K), namely a low loading (2–6 wt%, Fig. 2C,D), a moderate loading (20–60 wt%, Fig. 2E,F), or a high rubber deposition (120–130 wt%, Fig. 2I). The highest concentrated solution (175–200 wt% of rubber loading, Fig. 2J) causes the complete mat porosity loss in favour of an interfibrous film formation; however, this condition would remove the advantage of using a highly porous medium. The use of NBR solutions with a rubber concentration below 1.0 wt% (Fig. 2C,D) leads to a rubber loading not higher than 5–6 wt%, without producing any morphological difference with respect to the Nylon 66-unmodified mat (Fig. 2A). The rubber loaded on the Nylon nanofibers is clearly related to the viscosity of the impregnating solutions, up to saturation at the highest concentrations that, in facts, corresponds to the above discussed bulk film formation, as visible in Fig. 2K.

### Evaluation of Mode I interlaminar fracture toughness

The interlaminar fracture toughness of the nanomodified CFRPs was assessed via Double Cantilever Beam (DCB) tests. During the test, the specimen beams are subjected to a perpendicular load with respect to the crack propagation plane (Mode I loading mode). The resulting energy release rate (G_I_), calculated from the delamination test data, can be associated with two different crack propagation stages: the initiation stage (G_I,*C*_), in which the delamination onset starts from the artificial crack triggered by the Teflon film inserted during lamination, and the propagation stage (G_I,*R*_) resulting from subsequent crack advancements.

### DCB tests on low-amount NBR-impregnated mats

Two impregnating NBR solutions (0.2 wt% and 1.0 wt%) were chosen to evaluate the impact of low NBR loadings on Mode I interlaminar fracture toughness. The investigation has been carried out using both Nylon 66 membrane types (NyAcF and NyTFA), having an average mat thickness of 40 and 90 µm, and equivalent to grammages in the 10–11 and 25–27 g/m^2^ range, respectively (see Table S1 for details). The good agreement between grammage and thickness in the NyAcF and NyTFA systems stems from the similar fiber diameter attained, as demonstrated by a previous work investigating the grammage-thickness relationship^44^.

**Figure 3:**
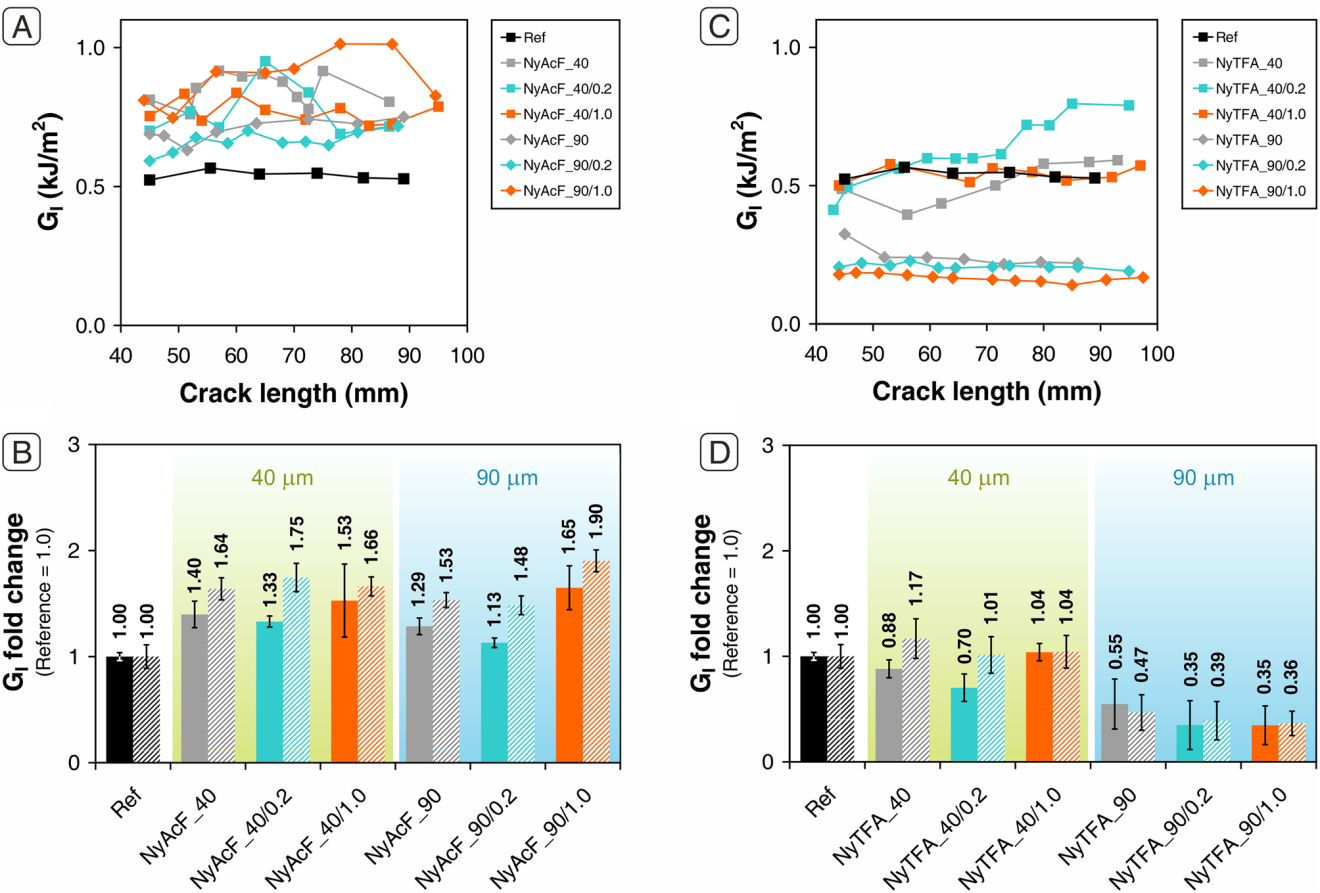
DCB test results of laminates nanomodified with NyAcF and NyTFA mats impregnated using 0.2 wt% (cyan points and bars) and 1.0 wt% (orange point and bars) NBR solutions: (**A**, **C**) *R*-curves of one representative specimen for each tested sample; (**B**, **D**) average G_I_ fold change (bars are expressed as the relative variation of the value with respect to the reference sample, whose value is set as 1.0).

Representative *R*-curves (fracture toughness vs. delamination length curves) are shown in Fig. 3A,C for CFRPs modified with NyAcF and NyTFA, respectively. At first glance, some significant differences can be highlighted, with some mats able to improve G_I_ significantly while others noticeably worsen it. In particular, G_I_ results show a general positive action of NyAcF mats against delamination (Fig. 3A,B). Unmodified NyAcF nanofibers can already enhance G_I_ between 53 and 64%, irrespective of the mat thickness. By contrast, plain NyTFA mats cause a reduction of the interlaminar performance when a 90 µm membrane is interleaved, leading to a halved G_I_ (Fig. 3C,D).

When considering the cases with a positive impact of the nanofibrous membrane interleave (NyAcF), the addition of thin NBR coatings to the fibers does not further improve the interlaminar fracture toughness. Concluding, low and very low rubber loadings do not further enhance G_I_ significantly, with only a few cases displaying an additional positive contribution compared to the plain Nylon membrane, such as NyAcF_40/0.2, NyAcF_40/1.0 and NyAcF_90/1.0 (up to + 65% in G_I,*C*_ and + 90% in G_I,*R*_). Moreover, thicker nonwovens (NyAcF_90 series) give almost the same reinforcing action as thinner ones (NyAcF_40 series). Even when plain Nylon 66 fibers behave poorly (NyTFA series), the rubber does not enhance the reinforcing action of plain polyamide. Moreover, the thickness of the membrane plays a disruptive role in this case, with both plain and rubber-loaded 90 µm NyTFA mats all causing a severe G_I_ reduction (up to − 65%); in contrast, the 40 µm ones have an almost neutral effect on the delamination behaviour.

Since high-thickness membrane production requires additional processing time, besides the increase of final weight and dimensions of the nanomodified CFRPs, further investigations are carried out by only integrating mats with an outmost 40 µm thickness and by loading higher rubber amounts.

### DCB tests on moderate NBR-impregnated mats

**Figure 4:**
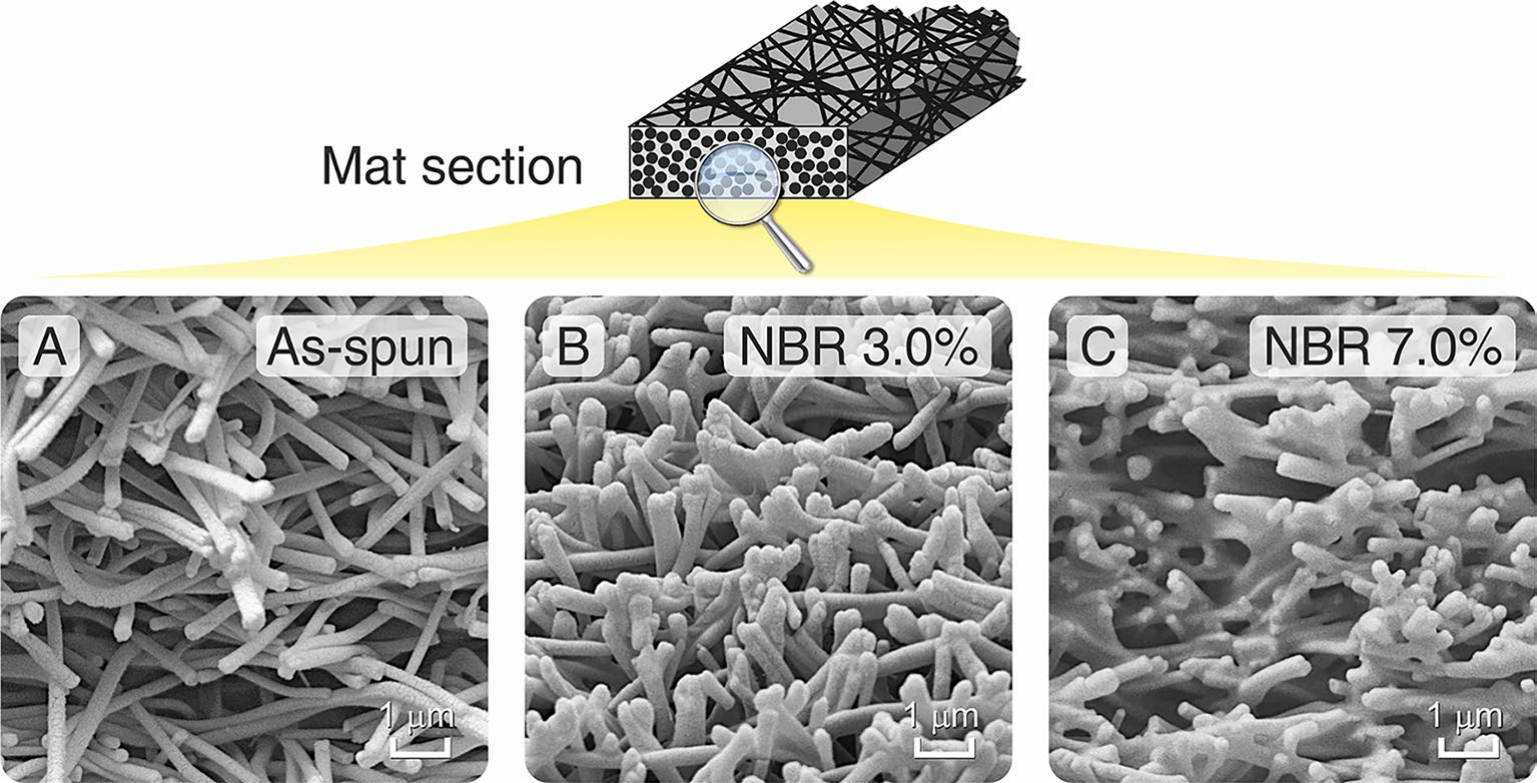
Cross-section SEM images of representative mats: (**A**) as-spun, and impregnated with NBR solution at (**B**) 3.0 wt% and (**C**) 7.0 wt% (NyTFA membranes).

The results presented in the previous section show three main facts: (i) NyAcF and NyTFA mats, even though made of the same Nylon 66, when interleaved in CFRPs behave differently against interlaminar fracture toughness; (ii) mats with a high thickness, even when impregnated with rubber, lead to results comparable to 40 µm mats or worse than the reference laminate; (iii) a thin NBR coating does not significantly enhance G_I_ with respect to plain Nylon 66 nanofibers. These findings point to the fact that the bridging mechanism is predominant due to the poor rubbery fraction and that the thermoplastic nanofibers performance governs the overall reinforcing effect in these conditions. Possible explanations for the different action of NyAcF and NyTFA mats (point i) will be discussed later. For the reasons explained in points ii and iii, mats with a maximum 40 µm thickness and a higher NBR loading were investigated: 10, 20, and 40 µm membranes were thus impregnated with 3.0 wt% and 7.0 wt% NBR solutions. Their viscosity should still guarantee sufficient retention of the mat porosity to ensure its effective impregnation by the prepreg-delivered matrix, as confirmed by surface SEM images (Fig. 2G,I). The observation of the mat across its thickness (section view, Fig. 4) reveals that using the 3.0 wt% NBR solution slightly affects the mat morphology, which is highly reminiscent of the plain Nylon 66 one, except for a more compact aspect. By contrast, using the 7.0 wt% impregnating solution has a more relevant impact on the nanofibrous membrane, reducing the voids as result of the retention of some NBR fraction between nanofibers. The different NBR loadings, already appreciable from SEM observations, are confirmed by the mats’ grammage and loaded rubber, as reported in Table 1.

These Nylon/NBR mats have been chosen to compare the anti-delamination performance delivered by relatively high NBR loadings (40–300 wt% with respect to plain polyamide nanofibers), still avoiding the formation of a compact film as it happens when impregnating with the highest concentrated rubber solution (10.0 wt%, Fig. 2J).

**Figure 5:**
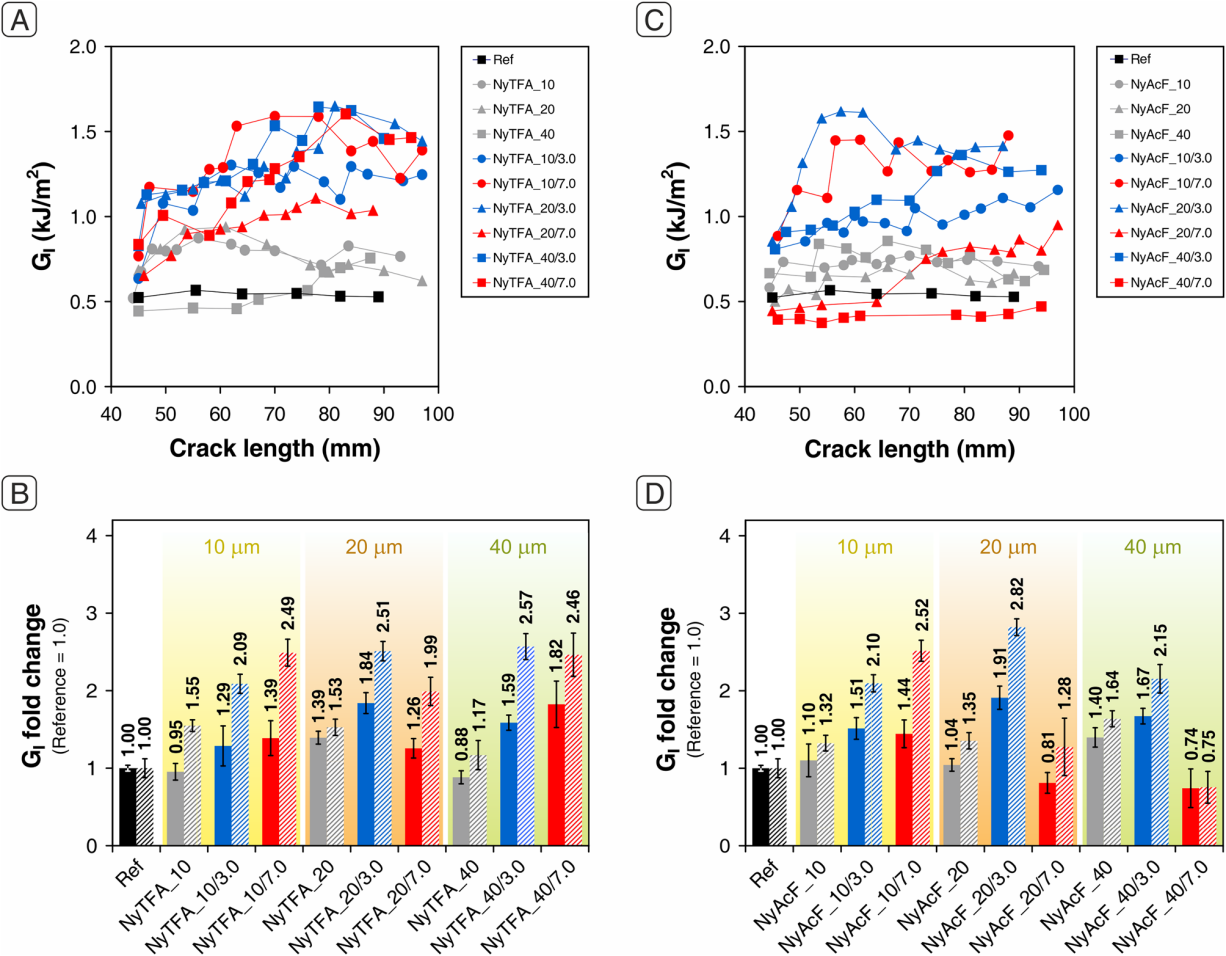
DCB test results of laminates nanomodified with NyTFA (**A**, **B**) and NyAcF (**C**, **D**) mats impregnated using 3.0 wt% (blue points and bars) and 7.0 wt% (red points and bars) NBR solutions: (**A**, **C**) *R*-curves of one representative specimen for each tested sample; (**B**, **D**) average G_I_ fold change (bars are expressed as the relative variation of the value with respect to the reference sample, whose value is set as 1.0). The performance of laminates reinforced with plain Nylon 66 mats are reported in gray.

Regarding the DCB tests on NyTFA nanomodified laminates (Fig. 5A,B and Table S2 in Supporting Information), two main considerations can be drawn. The first one is that below 40 µm the membrane thickness affects the energy associated with the interlaminar fracture. Indeed, plain NyTFA mats show a limited (but significant) maximum enhancement of G_I_ only when 20 µm mat is integrated (40–50% of G_I_ improvement for NyTFA_20). Lower or higher thicknesses do not almost modify G_I_, while in the already discussed case of NyTFA_90 there is a severe G_I_ reduction. The second consideration is that rubber-coated nanofibers with a significant amount of NBR loaded, above 40 wt% (Table 1), show an appreciable reinforcing effect (up to + 84% in G_I,*C*_ and + 157% in G_I,*R*_). In this case, indeed, the negative performance highlighted above for NyTFA series changes into a positive contribution against delamination.

No significant differences are evidenced between the two different NBR loadings, making the use of membranes with the lower rubber content preferable to limit the final laminate weight increase. The best overall delamination performance is thus achieved by using NyTFA_20/3.0 mat. Even more promising results are obtained using intermediate rubber-loaded NyAcF mats (Fig. 5C,D and Table S3 in Supporting Information). All the membranes impregnated with a 3.0 wt% NBR solution show a high delamination hindering action and a significant performance improvement with respect to the plain NyAcF mats. The best results are obtained by integrating the NyAcF_20/3.0 mat: + 91% in G_I,*C*_ and + 182% in G_I,*R*_.

**Figure 6:**
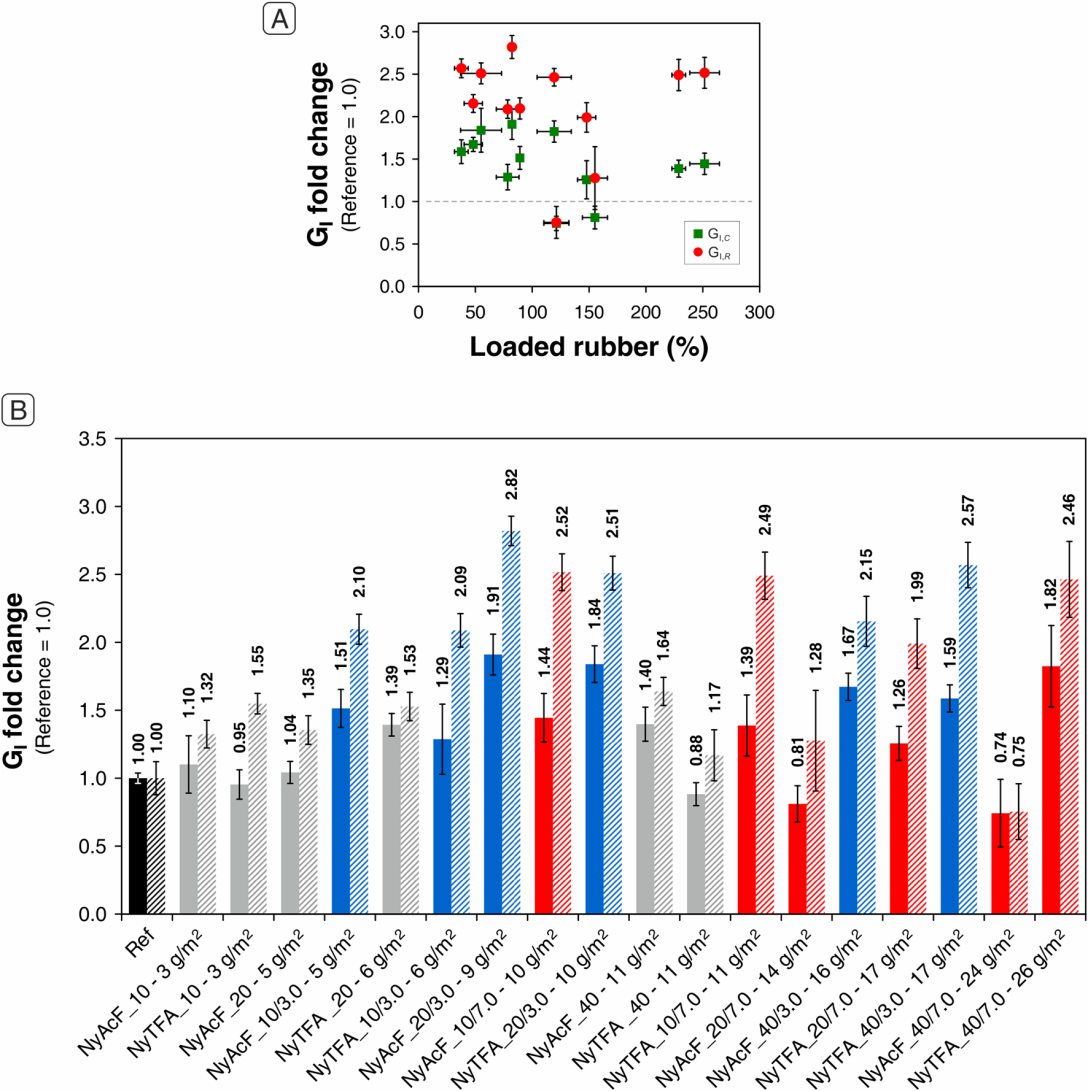
(**A**) Relation between G_I_ fold change and loaded rubber on Nylon 66 mats impregnated with NBR solutions at 3.0 wt% and 7.0 wt%. The performance of the reference sample is set at 1.0. (**B**) Relation between G_I_ fold change and final grammage of mats impregnated with NBR solutions at 3.0 wt% (blue bars) and 7.0 wt% (red bars). The performance of laminates reinforced with NyAcF and NyTFA plain mats are reported for comparison (gray bars).

Different behaviour is shown by NyAcF mats impregnated with a 7.0 wt% NBR solution. In this case, only the thinnest mat benefits from rubber impregnation (+ 44% in G_I,*C*_ and + 152% in G_I,*R*_), while the others perform worse than the reference laminate. Definitely, they behave similarly to some NyTFA mats, which act as a releasing film when integrated into the epoxy laminate. Figure 6A displays the G_I_ fold change in relation to the percentage of loaded rubber on the Nylon 66 mat. The achieved performance cannot be exclusively explained by considering the rubber percentage loaded on the nanofibrous mat. Generally, loadings below 100% provide G_I_ enhancements of 50–150%, regardless of the mat type (NyTFA or NyAcF) and mat thickness. However, it is impossible to affirm that major NBR loadings always lead to worse performance. For example, 10 µm mats, even though having an NBR loading > 200%, lead to + 30–50% in G_I,*C*_ and + 110–150% in G_I,*R*._ Probably, the presence of a high rubber percentage (but not very high at absolute values) can compensate for the poor effectiveness of the thermoplastic-only mat, which is not thick enough to prevent crack propagation. On the contrary, when dealing with medium and high thickness membranes, even an NBR loading percentage not exceptionally high can generate lower enhancements, or even G_I_ performance worse than the unmodified CFRP, as NyAcF mats with 20 and 40 µm thickness. However, it is neither possible to assume that low total mat grammages, i.e., considering the grammage deriving from both Nylon 66 nanofibers and the NBR coating, always give the best results (Fig. 6B).

Grammages in the 5–11 g/m^2^ range display a good reinforcement action toward Mode I delamination. Just only 5 g/m^2^ mat (NyAcF_10/3.0) allows to increase G_I,*C*_ of 51% and G_I,*R*_ of 110%, while almost doubling the final grammage, NyAcF_20/3.0 mat, gives the best absolute results: + 91% in G_I,*C*_ and + 182% in G_I,*R*_. It seems, in fact, that the final mat performance derives from a complex interplay of many different factors based on the total amount of materials, their actual composition (relative fractions of thermoplastics and rubber), morphology, and characteristics of the electrospun solution. This fact makes it difficult to extrapolate the most promising “set” of parameters leading to an optimized formulation, preventing them from being clearly identified and separated independently from one another.

By comparing the Mode I delamination of rubbery-reinforced laminates, the best-balanced performance is achieved by integrating 20 µm mats impregnated with NBR solution at 3.0 wt%, regardless of the NyAcF or NyTFA membrane type. Indeed, the G_I,*C*_ and G_I,*R*_ improve by 80–90% and 150–180%, respectively, still maintaining a low overall mat grammage of 9–10 g/m^2^.

**Figure 7:**
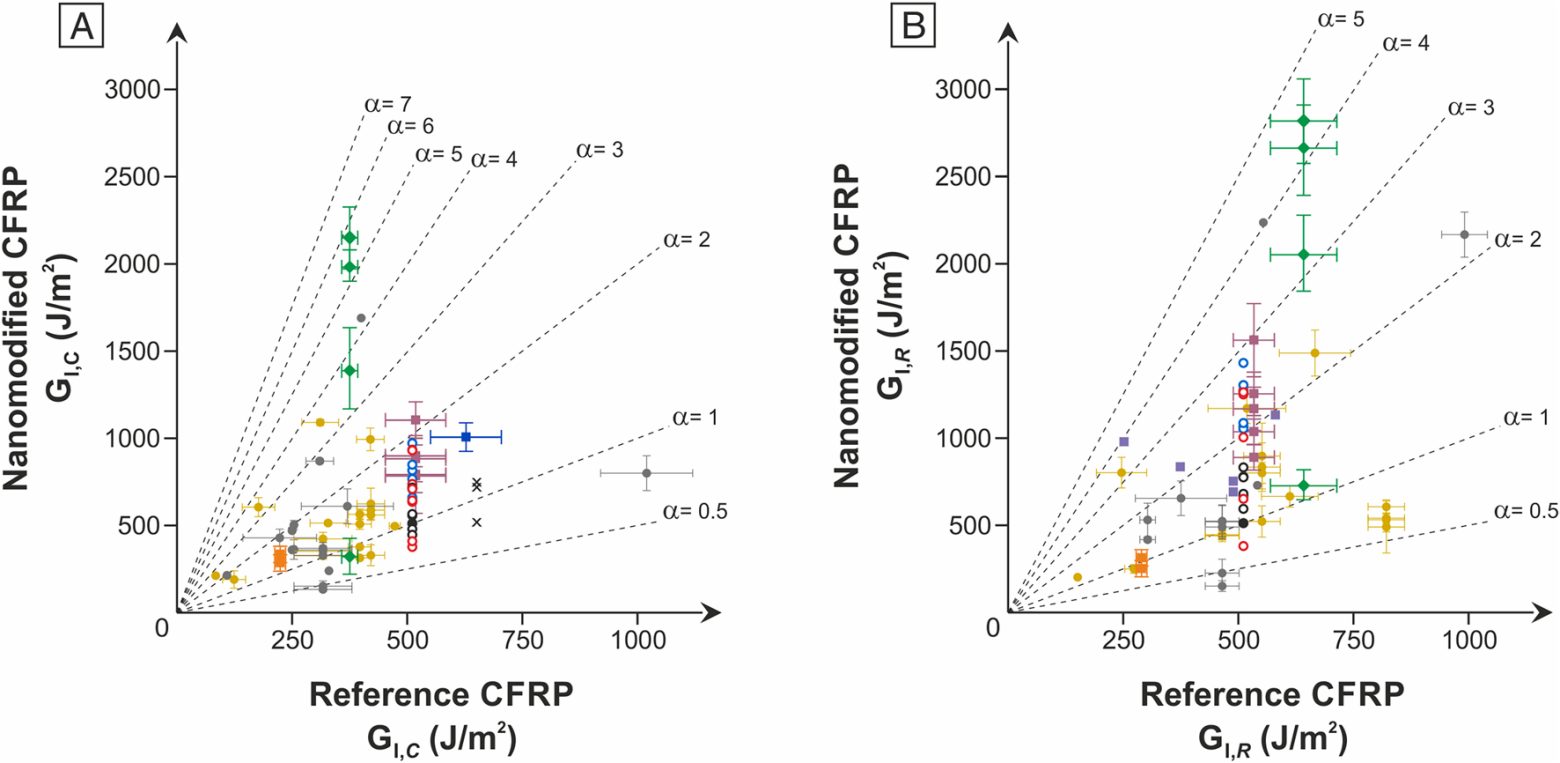
Comparison of Mode I energy release rate of tested composites with literature data: (**A**) G_I_ initiation, and (**B**) G_I_ propagation. Legend: circles identify the laminates tested in the present work (solid black circle, reference; black circles, plain Nylon 66 mats; blue circles, mats impregnated with the 3.0 wt% NBR solution; red circles, mats impregnated with the 7.0 wt% NBR solution); yellow polyamide nanofibers^26^; green, NBR/PCL nanofibers^32^; spent fucsia, NBR/Nomex nanofibers^33^; dark blue, PEO nanofibers^46^; grey, “other” nanofiber types^26^.

The results achieved by interleaving such rubber/thermoplastic nanofibers reveal an effective boost of the CFRP interlaminar properties. Literature data^26,32,33,36–40,45–50^ regarding polyamide nanomodification with Nylon 6 and 66 generally report improvements in the Mode I fracture toughness in the 25–60% range (Fig. 7), with few exceptions in both directions (lower and higher G_I_ values). Such improvements align with those recorded upon modification with plain Nylon 66 nanofibers (up to + 64% using NyAcF mats). Therefore, the performance gap between as-spun polyamide nanofibers and rubber-coated one can be entirely attributed to the favourable action of NBR, as previously found^33^ for Nomex nanofibers mixed with the same rubber.

A comparison of the performance improvements delivered by the proposed rubber/thermoplastic nanofibers with similar systems is not possible since, to the best of the authors’ knowledge, the use of rubber as a “coating” for thermoplastic nanofibers is unprecedented. The most similar works for a rough comparison are (i) Nylon/PCL core–shell nanofibers^34,35^, and (ii) NBR/Nomex ones^33^. In the first case, the PCL shell, added to the polyamide via core–shell electrospinning technique, allows achieving an increment of G up to + 65%. In the latter, mixed NBR/Nomex nanofibers are obtained via the single-needle electrospinning technique of an emulsion of the two polymers which, under the specific process conditions, self-assembly. In this case, a “continuous arrangement” of the polyaramide surrounded by the NBR is obtained, similarly to what happens by carrying out a core–shell electrospinning. The NBR presence is fundamental to achieve an excellent reinforcing action (up to + 180% in G_I_), while the integration of plain Nomex nanofibers, acting as a release film, strongly favours the composite delamination (– 70% in G_I_ with respect to the commercial unmodified laminate).

### Crack path and delamination surfaces analysis

**Figure 8:**
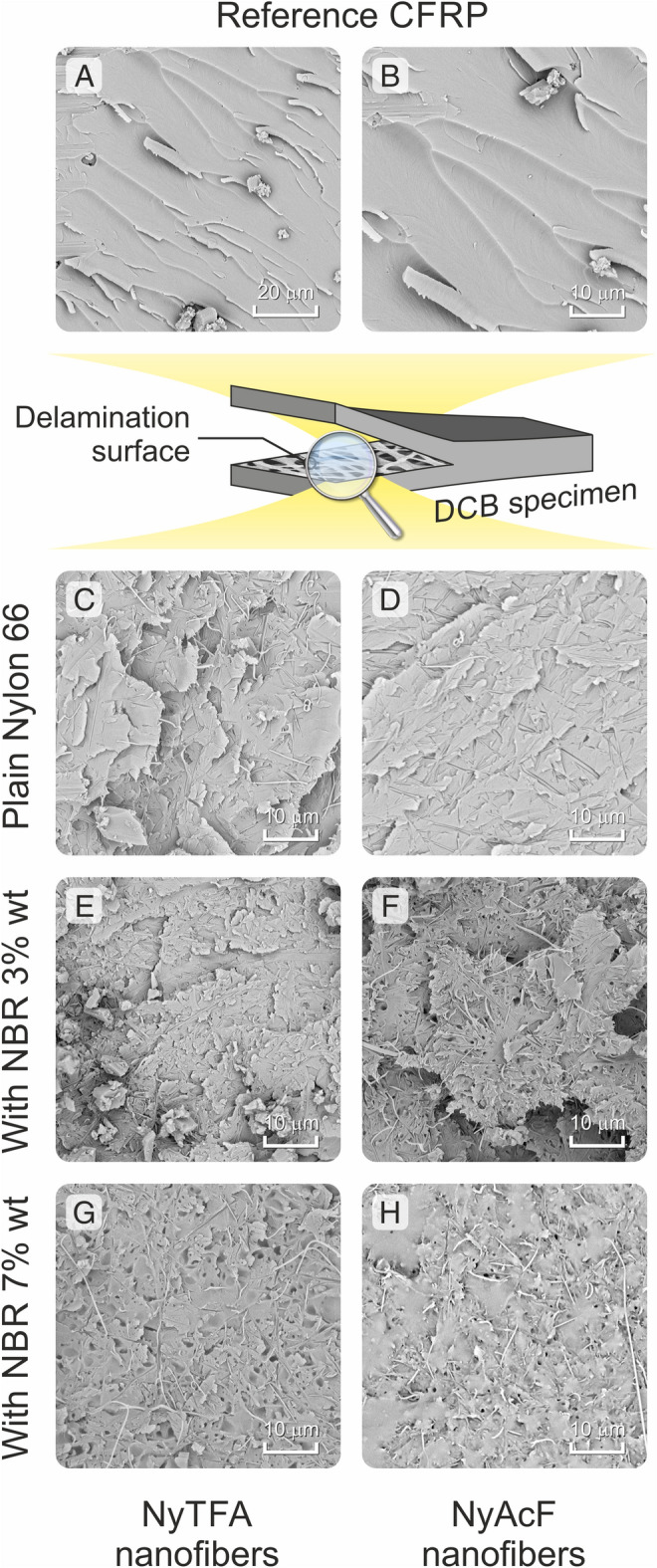
SEM micrographs of delamination surfaces after DCB test: (**A**, **B**) reference CFRP; (**C**–**H**) nanomodified CFRPs (1st column, with NyTFA mats; 2nd column, with NyAcF mats). For the nanomodified laminates, representative images of selected samples are reported. Displayed samples: (**C**) NyTFA_20; (**D**) NyAcF_20; (**E**) NyTFA_20/3.0; (**F**) NyAcF_20/3.0; (**G**) NyTFA_10/7.0; (**H**) NyAcF_40/7.0.

The SEM investigation of delamination surfaces, taken after the DCB test, is helpful to visualize the effect of nanofibers on the fracture morphology with respect to the reference laminate. The surface of the unmodified CFRP is characterized by wide and smooth matrix flat planes accounting for the brittle fracture of epoxy resin (Fig. 8A,B).

Analysing the delamination surfaces of nanomodified composites, it can be noticed that Nylon 66 nanofibers are still visible (Fig. 8C–H), as expected based on their thermal properties. Indeed, the curing temperature does not exceed the polyamide melting temperature (135 °C vs 266 °C, as assessed via DSC analysis). However, there is a difference between samples reinforced with plain Nylon 66 nanofibers (Fig. 8C,D) and with rubber-loaded ones (Fig. 8E–H). In the latter, the surrounding matrix is toughened, as testified by the presence of plastic deformation. This behaviour becomes more evident for membranes impregnated with the most concentrated (7 wt%) NBR solution (Fig. 8G,H). Moreover, the flat planes, still reminiscent of brittle matrix fracture, are completely lost, as already observed when the nanomodification is carried out through polyethylene oxide (PEO)^46^, NBR/PCL blend^32^ and NBR/Nomex mixed^33^ nanofibers. By contrast, such planes are visible when the mats are impregnated by the 3.0 wt% NBR solution, highlighting that the matrix toughening is lower.

### On the different behaviour of NyTFA and NyAcF mats at contrasting delamination

It has been previously stated that the overall behaviour of NBR-impregnated Nylon 66 nanofibers is extremely hard to analyse in terms of specific operational parameters contribution. It is nonetheless true that Mode I delamination tests highlight that the reinforcing action of polyamide nonwovens depends on the starting electrospinning solution characteristics. In general, NyAcF mats perform better than NyTFA ones in the present conditions; this statement is, in fact, true when plain nanofibrous mats are interleaved in CFRPs. Their different behaviour may be explained by the potential different thermal and mechanical properties of NyAcF and NyTFA mats achieved during the electrospinning process.

**Figure 9:**
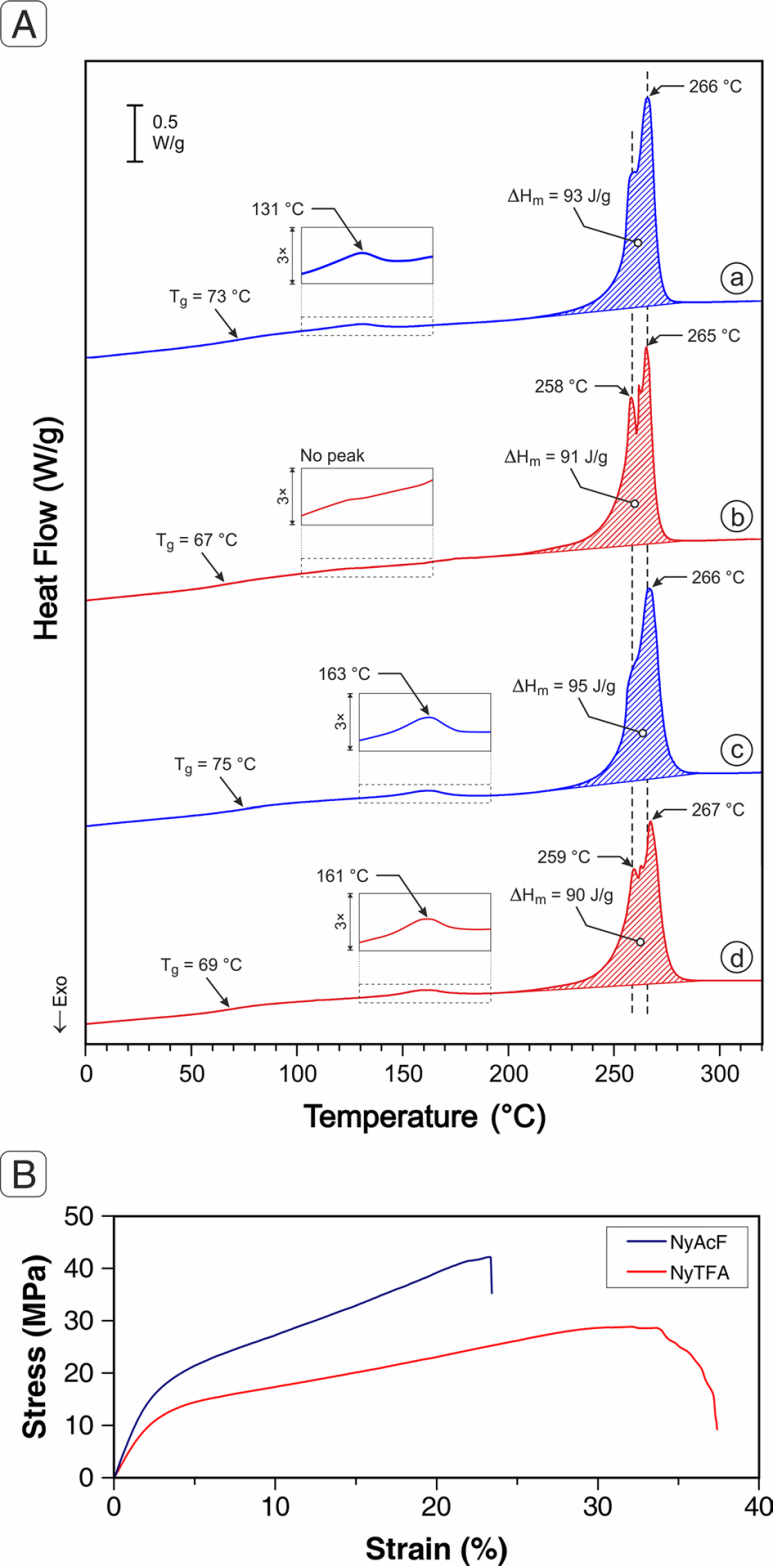
(**A**) DSC analysis of plain NyAcF (blue curves, a, c) and NyTFA (red curves, b, d) nanofibrous mats: (a, b) as-spun nanofibers; (c, d) after thermal treatment simulating the composite curing cycle. Enlargements (3 ×) of the region indicating the so-called Rigid Amorphous Fraction (RAF) are displayed for a better clarity. (**B**) Stress–strain test of plain Nylon 66 mats: comparison of tensile curves of NyAcF and NyTFA nanofibrous membranes.

An attempt at explaining the observed differences has been addressed by evaluating the thermal behaviour of the samples: DSC analysis (Fig. 9A, thermograms a,b) reveals, at first glance, for the two electrospun polymers, a stepwise transition, and a complex endothermic peak: they account for the glass transition (*T*_g_) and crystal phase melting, respectively. Recorded data show that the as-spun NyAcF mat displays a higher *T*_g_ with respect to the one of NyTFA membrane (73 °C vs 67 °C), while the degree of crystallinity associated with the endotherm is comparable in both cases (χ_c_ = 46–47%, considering a ΔH_m, 100% cryst._ = 196 J/g^51^). Moreover, while both melting endotherms show a main high-T peak around 266 °C, a lower temperature signal (258 °C) is clearly visible in the NyTFA mat thermogram, but it is decidedly less pronounced for NyAcF nanofibers, being simply a shoulder of the main peak. Furthermore, when focussing on the 130–190 °C region, which should be void of any signal, in NyAcF, a weak peak is detected, which instead lacks in the NyTFA thermogram.

Such a transition has been associated in the literature with the so-called Rigid Amorphous Fraction (RAF)^52,53^, a rather anisotropic region where hydrogen bonds between amide groups randomly but frequently form even without the ordered arrangement of the crystalline phase. Typically, hydrogen bonds form regularly spaced along a single direction owing to a correct arrangement of CO and NH groups in neighbouring chains. In the RAF, H-bond formation seems indeed to be favoured in the presence of macromolecular prevailing alignment, such as the fibrous arrangement, and might bear along as a consequence of the presence of an additional amount of intermolecular H-bonds. Even if these interactions do not result in an increased crystal phase, they might still help the intrinsic mechanical performance of the material, improving, in turn, the nanofiber-bridging ability. Another effect due to intermolecular H-bonds could be that the amide groups are kept “captive,” reducing their availability to face out the fiber structure. As a consequence, the surface energy of the nanofibers changes, affecting their ability to interact with the epoxy matrix. Indeed, it was found that nanofibers made of high-performance Nomex nanofibers promote CFRP delamination due to low adhesion with epoxy resin^33^.

This hypothesis is extremely difficult to test directly on the substrate since the nanofibrous arrangements do not allow for a test of the intrinsic material surface properties, while it is exactly the fiber spinning that seems at the basis of the RAF formation. Overall, the previous assumptions on thermal properties may imply an improved NyAcF mat mechanical performance with respect to the NyTFA one. Previously, it was demonstrated that the number of nanofiber crossings, related to the nanofiber diameter, also affects the mat mechanical properties: the lower the fiber diameter, the higher the nanofiber crossings and the tensile properties^44^. In the present case, however, such an effect should be negligible, given that the two different nanofibrous mat types have nanofibers with comparable diameters and grammages. Nonetheless, tensile tests confirm (Fig. 9B and Table S4 in Supporting Information) that the NyAcF mat displays an improved elastic modulus and strength (+ 43% and + 35% with respect to NyTFA one, respectively), with the overall toughness of both mat types which is comparable.

Since nanofibers undergo a thermal treatment during the curing process, which is above the glass transition, the process might somehow affect their thermal behaviour. Membranes were thus investigated after applying a simulated curing cycle. The DSC thermograms recorded after curing cycle simulation (Fig. 9A, thermograms c,d) show almost no relative difference between the thermal properties of the two nanofibrous mat types, besides a slight improvement of both *T*_g_ and crystal phase extent (χ_c_ = 46–49%). The annealing, carried out in a condition providing some extent of mobility of the amorphous chains, might help interaction in between the prevailingly oriented macromolecules, and indeed, a temperature upshift of the RAF transition in NyAcF occurs (131 °C → 163 °C). Moreover, a small signal is now visible at 161 °C also in the NyTFA: it is worth to point out, however, that the latter is not associated to a boost in *T*_g_, which does not move upwards as much as NyAcF. This behaviour suggests that promoting H-bond formation after fibers are already shaped is not as effective as when it is formed during the fibers’ manufacturing in terms of overall materials performance, as it is, in fact, observed in the previous discussion. The nanofiber bridging efficacy is indeed related to the nanofibers’ mechanical properties, besides a good adhesion to the surrounding epoxy resin.

Summarising, the different reinforcing effects of NyTFA and NyAcF mats at contrasting delamination may derive from different (i) mats’ thermal behaviour, (ii) mats’ mechanical properties, and (iii) interaction of the polyamide with the epoxy resin delivered by the prepreg. Regarding point (i), DSC analysis reveals that there is only a slight difference between the polyamide membranes, limited to the NyAcF’s *T*_g_, which is higher than the NyTFA one. This is due to the RAF, which is also responsible for the NyAcF superior tensile properties, given that the degree of crystallinity and fiber diameters are similar to NyTFA nanofibers. The better mat mechanical properties may lead to a more effective “nanofiber bridging” (point ii); however, a different nanofiber-matrix adhesion (point iii) due to the negative effect of TFA solvent cannot be ruled out. In the literature, its use as a solvent/co-solvent for Nylon 66 electrospinning (except for two works from the authors^44,54^) is practically unexplored. Consequently, reports on Nylon 66 nanofibers electrospun from a TFA solution integrated into epoxy laminates are presently missing. Therefore, there are no reference literature data for comparing the current NyTFA membrane performance for contrasting delamination in epoxy-based composite laminates.

## Materials and methods

### Materials

Nylon 66, Zytel E53 NC010, kindly provided by DuPont, was dried in a stove at 110 °C for a minimum of 6 h before use. Nitrile Butadiene Rubber (NBR), carboxylated, Nipol 1072CGX, was purchased from Zeon Chemicals [68 mol% butadiene (Bu), 28 mol% acrylonitrile (ACN), 4 mol% methacrylic acid (MAA)]. Trifluoroacetic acid (TFA), formic acid, chloroform (CHCl_3_), and acetone, all reagent grade, were purchased from Sigma-Aldrich and used without further purification. Plain weave carbon fabric (200 g/m^2^) in epoxy matrix prepreg (GG204P IMP503Z-HT) for composite lamination was supplied by G. Angeloni s.r.l. (Venezia, Italy).

### Solutions preparation, nanofibrous mats production, and their characterization

Nylon 66 solutions were prepared using two different solvent systems. Ny10 solution (10 wt% in polymer) was made dissolving in sealed vials polyamide pellets in formic acid/CHCl_3_ 1:1 wt (55:45 vol.) under magnetic stirring and mild heating (maximum 50 °C) until complete polymer dissolution. Ny13 solution (13 wt% in polymer) was prepared using a different solvent system, made of TFA/formic acid/CHCl_3_ 11:55:34 wt (10:60:30 vol.).

NBR solutions at different concentrations (0.2, 0.5, 1.0, 2.0, 3.0, 4.0, 7.0, and 10.0 wt%) for the impregnation of Nylon 66 mats were prepared using acetone as a solvent, favouring rubber dissolution by magnetic stirring and mild heating (maximum 40 °C) until forming homogeneous solutions.

Nanofibrous mats were produced using a 4-needle electrospinning machine (Lab Unit, Spinbow) equipped with 5 mL syringes. Needles (internal diameter 0.84 mm, length 55 mm) were joined to syringes via Teflon tubing. Nanofibers were collected on a drum, rotating at a low speed (tangential speed 0.39 m/s), covered with poly(ethylene)-coated paper. Mats have final dimensions of approximately 30 × 40 cm. Nylon 66 mats with four different thicknesses (10, 20, 40, and 90 µm) were produced. NyAcF mats were obtained from Ny10 solution (electrospinning parameters: flow rate 0.50 mL/h, electric potential 24 kV, distance 15 cm, electrostatic field 1.6 kV/cm, temperature 23–25 °C, RH 22–25%). NyTFA mats were produced by Ny13 solution (electrospinning parameters: flow rate 0.80 mL/h, electric potential 23 kV, distance 11 cm, electrostatic field 2.1 kV/cm, temperature 23–25 °C, RH 22–25%). Mats impregnation was manually carried out by dropping with a Pasteur the impregnating NBR solution onto the Nylon 66 membrane; when detected, the excess of impregnating solution was removed by dabbing the mat with baking paper. Then they were placed in a desiccator under vacuum for at least 3 h, after which they were ready for integration within the laminates.

Nanofibrous mats are labelled as X_Y/Z, where X indicates the mat type (NyTFA or NyAcF), Y the mat thickness (10, 20, 40, or 90 µm), and Z the concentration of the impregnating NBR solution (if applicable).

Nanofibrous mats were analyzed by Scanning Electron Microscopy (SEM, Phenom ProX) to determine nanofibers' morphology. All analyzed surfaces were preliminarily gold-coated to make them conductive.

Differential Scanning Calorimetry (DSC) measurements were carried out on a TA Instruments Q2000 DSC apparatus equipped with an RCS cooling system. The nanofibrous mat samples (10 mg) were first heated to 100 °C for 15 min to remove humidity, then cooled down to – 50 °C and finally heated at 20 °C/min to 310 °C in a nitrogen atmosphere.

Viscosity measurements on NBR impregnating solutions were performed at 25 °C via a rotational viscometer (Haake Viscotester 7 plus).

Tensile tests of NyAcF and NyTFA mats were carried out using a Remet TC-10 universal testing machine equipped with a 10 N load cell, speed test 10 mm/min. Nanofibrous membranes were anchored in a paper frame (47 × 67 mm and 25 × 45 mm, outer and inner dimensions, respectively), gluing them with cyanoacrylate glue for better handling^44,55^. Effective specimen dimensions were 20 × 45 mm, (width) × (initial length), respectively. The paper frame was cut before starting the test. At least five specimens for each mat sample were tested. Load data were analyzed according to a reliable method based on the specimen mass load normalization instead of its cross-section area by applying the following equation for the stress (σ) evaluation:

1 $$\sigma ={\rho }_{m}\frac{F}{m}L$$

where *ρ*_*m*_ is the material density (in the present case, the density of Nylon 66, 1.14 g/cm^3^), *F* is the force, *m* is the specimen mass, and *L* is the specimen’s initial length. A full explanation of the normalization method is reported in Ref.^44^.

### CFRPs production and characterization

Specimens for the Mode I interlaminar fracture toughness evaluation via Double Cantilever Beam, DCB, tests were prepared via hand lay-up, stacking 14 prepreg plies, interleaving a single nanofibrous mat in the central interface, and adding a Teflon film as a crack trigger (Fig. S1, Supporting Information). A reference panel without interleaved nanofibrous mat was also produced for the sake of comparison. Uncured laminates underwent a preliminary treatment of 2 h at 45 °C under vacuum for better impregnation of nanofibers prior curing cycle in an autoclave (2 h at 135 °C, under vacuum, 6 bar external pressure, heating/cooling ramp of 2 °C/min). Composite panels maintain the nomenclature adopted for the nanofibrous mats; the unmodified laminate is labelled Ref. Details on laminates production and CFRP panels/specimens dimensions are reported in Supplementary Information.

DCB tests were carried out using a Remet TC-10 universal testing machine equipped with a 1 kN load cell. DCB specimens were tested at 5.0 mm/min crosshead separation rate. At least 3 specimens for each CFRP sample were tested.

The energy release rate for Mode I loading (G_I_, in J/m^2^), both at the initial and propagation stages (G_I,*C*_ and G_I,*R*_, respectively), was evaluated using Eq. 2^56^:

2 $${G}_{I}=\frac{3P\delta }{2ba}$$

where *P* is the load, *δ* the crosshead displacement, *b* is the specimen width, and *a* is the crack length. The G_R_ was evaluated considering a crack length range of 47–90 mm.

## Conclusions

The interleaving of thermoplastic nanofibrous mats is a well-established method to increase the interlaminar performance of thermosetting-based composite laminates. Recently, rubbery nanofibers demonstrated a remarkable ability to improve the interlaminar fracture toughness, suggesting their use to limit delamination.

The present work highlights the benefits of using elastomers for hindering delamination: Nylon 66 nanofibrous mats were impregnated with NBR, after their production via electrospinning, for producing rubber/thermoplastic membranes for hindering delamination in epoxy CFRP composites. The effect of two similar but different solvent systems for the polyamide electrospinning was investigated, as well as the mat thickness (grammage) and the amount of loaded rubber.

DCB tests reveal that the solvent system employed for producing Nylon 66 nanofibers affects the delamination behaviour of nanomodified composites: the best results are obtained when formic acid/chloroform is used (NyAcF mats, up to + 64% in G_I_), while the presence of TFA (NyTFA mats, up to + 53 in G_I_) leads to lower reinforcements and even to a performance worse than the unmodified CFRP. The addition of NBR is useful in many cases to further improve the interlaminar fracture toughness of plain Nylon 66 nanofibers; besides, it is also able to counterbalance the negative performance of the plain polyamide electrospun from the solvent system with TFA. The best results are obtained when interleaving medium-thickness and lightweight mats (20 µm, 9–10 g/m^2^) with 70–80 wt% of loaded rubber, achieving up to + 180% in G_I_.

The work demonstrates the ability of NBR to improve the delamination hindering of common and well-known polyamide nonwovens, paving the way for using NBR-coated Nylon 66 nanofibers as effective interleaves for localised interlaminar fracture toughness enhancement.

## Supplementary Information

Supplementary Information.

## Data Availability

All data generated or analysed during this study are included in this published article (and its Supplementary Information files).
