# Supplementary material for: Rubber-enhanced polyamide nanofibers for a significant improvement of CFRP interlaminar fracture toughness
Source: Sci Rep. 2022 Dec 11;12:21426. doi: 10.1038/s41598-022-25287-y (PMC9742143; doi:10.1038/s41598-022-25287-y)
Supplement: Supplementary file 1 — Supplementary Information. [file 41598_2022_25287_MOESM1_ESM.pdf]

Supporting Information

Rubber-enhanced polyamide nanofibers for a significant improvement of CFRP interlaminar fracture toughness

Emanuele Maccaferri[a,*], Matteo Dalle Donne[a], Laura Mazzocchetti[a,b,*], Tiziana Benelli[a,b], Tommaso Maria Brugo[b,c], Andrea Zucchelli[b,c], Loris Giorgini[a,b]

[a] *Department of Industrial Chemistry "Toso Montanari", University of Bologna, Viale Risorgimento 4, 40136 Bologna, Italy.*

[b] *Interdepartmental Center for Industrial Research on Advanced Applications in Mechanical Engineering and Materials Technology, CIRI-MAM, University of Bologna, Viale Risorgimento 2, 40136 Bologna, Italy.*

[c] *Department of Industrial Engineering, University of Bologna, Viale Risorgimento 2, 40136 Bologna, Italy.*

* Corresponding authors: emanuele.maccaferri3@unibo.it
laura.mazzocchetti@unibo.it

Production of CFRP laminates for DCB, ENF, 3PB and DMA tests

CFRP panels for DCB tests were produced via hand lay-up in an air-conditioned room (23-25 °C, RH 22-25%). The nanofibrous membranes were directly applied with their paper substrate onto the prepreg during the hand lay-up. Before the addition of the next prepreg ply, the supporting paper was removed. To favour the impregnation of the nanofibrous mat, uncured panels underwent a preliminary treatment of 2 h at 45 °C under vacuum before curing cycle in autoclave. Then, they were cured in an autoclave (2 h at 135 °C, under vacuum, 6 bar external pressure, heating/cooling ramp 2 °C/min).

CFRP panels for DCB tests (Figure S1), 140 × 190 mm, are constituted by 14 CFRP plies in total. Only the central interface was modified with the nanofibrous mat. A Teflon film was used as a crack trigger. The specimens were obtained by cutting out the panel; edge parts (minimum 15 mm) were discarded to avoid any inhomogeneity.

DCB specimens have the following final dimensions: 130 mm total length, 20 mm width (b), 45 mm initial crack length (a_0). Aluminum blocks were fixed to the DCB specimen with epoxy resin glue to anchor the specimen on the testing machine.

ENF specimens had the following dimensions: 160 mm total length, 20 mm width (b), 45 mm initial crack length (a_0).

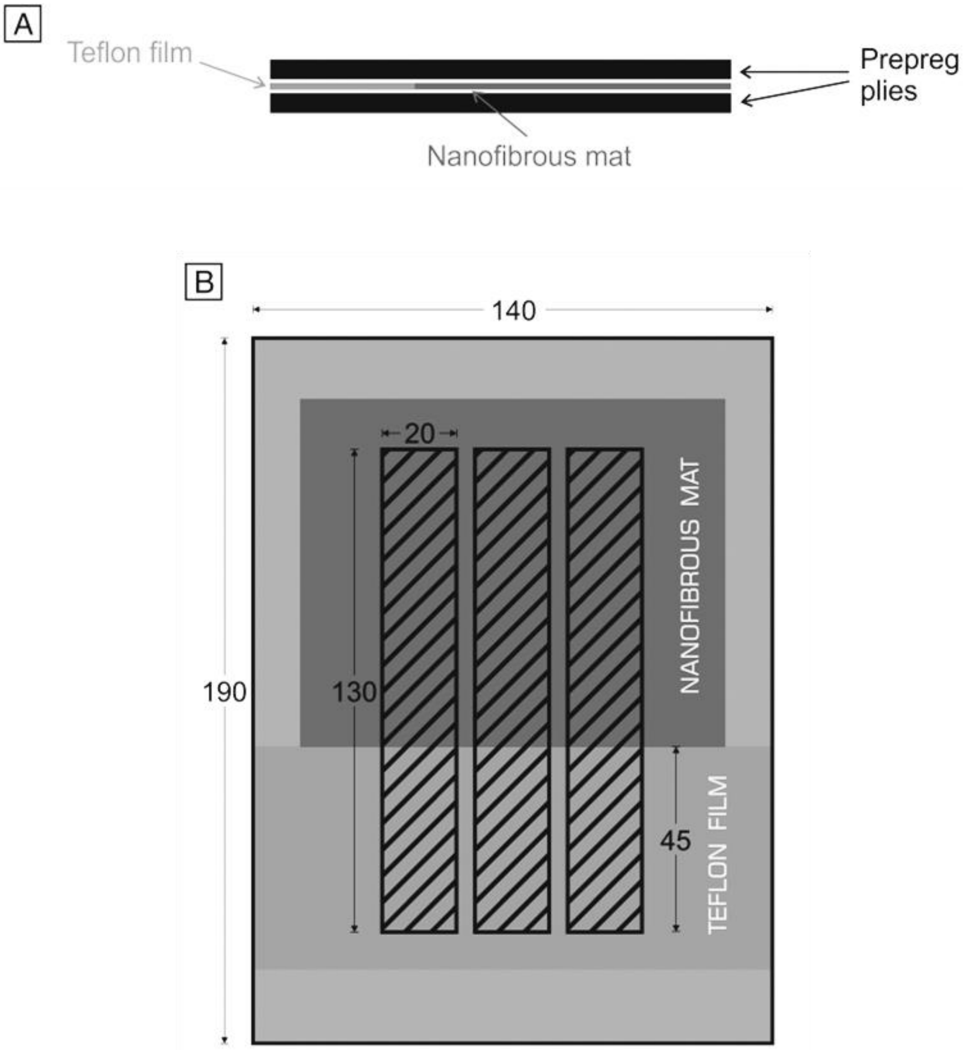

Fig. S1 – CFRP panels for characterization of delamination resistance: laminate section view (A) and dimensions (in mm) of panels and specimens for DCB test (B).

Table S1 – Mat characteristics before and after their impregnation with NBR solutions at 0.2% wt and 1.0% wt.

Nanofibrous mat	Nylon mat grammage (g/m^2)	Loaded rubber (% wt)		Ratio B/A
		(A) with 0.2% wt NBR solution	(B) with 1.0% wt NBR solution	
NyAcF_40	10.8 ± 0.7	2-5	14-24	≈ 5
NyAcF_90	25.7 ± 0.8	4-7	20-25	≈ 4
NyTFA_40	11.4 ± 0.8	2-4	15-22	≈ 6
NyTFA_90	26.6 ± 0.9	4-7	18-27	≈ 4

Table S2 – DCB test results of CFRPs nanomodified with NyTFA membranes.

**CFRP sample**	**Max. Load** (N)	$\mathbf{G_{I,\mathit{C}}}$ (J/m^2)	$\mathbf{G_{I,\mathit{R}}}$ (J/m^2)
C-Ref	41.1 ± 1.2	508 ± 19	506 ± 62
NyTFA _10	49.7 ± 1.0	485 ± 52	784 ± 59
NyTFA_10/3.0	53.4 ± 3.3	654 ± 169	1057 ± 130
NyTFA_10/7.0	57.8 ± 2.5	705 ± 158	1262 ± 241
NyTFA _20	47.8 ± 1.3	708 ± 59	773 ± 81
NyTFA_20/3.0	60.0 ± 0.9	935 ± 126	1270 ± 158
NyTFA_20/7.0	49.2 ± 7.5	638 ± 80	1007 ± 184
NyTFA _40	39.8 ± 0.9	448 ± 38	591 ± 111
NyTFA_40/3.0	57.3 ± 1.7	806 ± 26	1300 ± 217
NyTFA_40/7.0	55.2 ± 7.7	927 ± 277	1247 ± 348

Table S3 – DCB test results of CFRPs nanomodified with NyAcF membranes.

**CFRP sample**	**Max. Load** (N)	**$G_{I,C}$** (J/m^2)	**$G_{I,R}$** (J/m^2)
C-Ref	41.1 ± 1.2	508 ± 19	506 ± 62
NyAcF_10	45.0 ± 4.4	560 ± 118	670 ± 68
NyAcF_10/3.0	52.4 ± 2.5	769 ± 107	1061 ± 117
NyAcF_10/7.0	55.3 ± 2.8	734 ± 131	1273 ± 172
NyAcF_20	41.6 ± 1.3	530 ± 43	685 ± 72
NyAcF_20/3.0	59.2 ± 2.3	968 ± 230	1427 ± 151
NyAcF_20/7.0	34.6 ± 2.2	412 ± 55	646 ± 239
NyAcF_40	51.8 ± 3.9	710 ± 89	829 ± 86
NyAcF_40/3.0	52.0 ± 1.0	850 ± 43	1090 ± 201
NyAcF_40/7.0	34.7 ± 4.1	377 ± 94	382 ± 78

Table S4 – Tensile properties of plain Nylon 66 mats.

Membrane type	E [MPa]		σ_{max} [MPa]		$\varepsilon_{\sigma max}$ [%]		U [J cm^{-3}]	
	Mean ± SD	CV [%]	Mean ± SD	CV [%]	Mean ± SD	CV [%]	Mean ± SD	CV [%]
NyAcF	723 ± 98	13	42 ± 6	6	22 ± 2	9	7.9 ± 1.2	15
NyTFA	512 ± 61	12	32 ± 2	6	34 ± 2	5	8.3 ± 0.6	7
